# Monitoring Sarcopenia With Incretin Receptor Activator Treatment

**DOI:** 10.1111/1753-0407.70117

**Published:** 2025-06-18

**Authors:** Zachary T. Bloomgarden

**Affiliations:** ^1^ Department of Medicine, Division of Endocrinology, Diabetes and Bone Disease Icahn School of Medicine at Mount Sinai New York New York USA

Obesity prevalence has increased over many decades, with one quarter of the adult population projected to be obese by 2035 [1]. The glucagon‐like peptide 1 receptor agonists (GLP‐1RA) and related agents are associated with effective and sustained weight loss and show evidence of reduction in a myriad of cardiovascular (CV) and renal outcomes [2], as well as improving metabolic, hepatic, respiratory, and musculoskeletal complications of obesity. An increasing concern, however, is whether concomitant reduction in skeletal muscle mass (SMM) with fat mass may have adverse consequences [3].

While there is normally a decrease in SMM of 10%–15% from age 20 to 80 [4], excessive loss in muscle mass and the accompanying reduction in strength is associated with increased mortality, both overall and that due to a variety of separate conditions, whether measured based on bioelectrical impedance [5], self‐reported walking limitation [6], or reduction in maximum handgrip strength [7]. In the National Health and Nutrition Examination Survey, 17%, 26%, and 31% of obese people aged 60–70, 70–80, and ≥ 80 had evidence of sarcopenia based on the appendicular lean mass‐to‐BMI ratio using dual‐energy X‐ray absorptiometry [8]. In further evidence of the prevalence of sarcopenic obesity, reduced handgrip strength and walking speed was present in 30%, 62%, and 89% of obese persons at ages 45–60, 61–80, and ≥ 80 in the Longitudinal Aging Study in India [9].

Across studies of dietary weight loss, GLP‐1RA, and bariatric surgery, the relationship between the fraction of body weight lost and the fraction of lean body mass lost is linear, with somewhat limited data showing approximately 5% such loss for every 10% body weight reduction [10]. In a meta‐analysis of 36 studies of bariatric surgery, the mean decrease in SMM averaged 7.8 kg at 12 months, with the first, second, third, and fourth quartiles of decrease in SMM 11.0, 9.1, 6.7 and 4.4 kg, respectively [11].

About one in eight adults say they have at some point taken a GLP‐1RA, with about half of these currently taking the medications [12]. It is, then, important to ask whether this widely used treatment is associated with a reduction in SMM. These agents on average are associated with an improvement in physical function [13], and some have suggested that clinical trials have not shown GLP‐1RA to be associated with “clinically relevant” skeletal muscle loss [14]. However, clinical trial populations differ in important ways from individuals in clinical treatment [15, 16, 17], the latter typically older with greater degrees of frailty and multiple comorbidities. The largest meta‐analysis allowing insight into the effect of GLP‐1RA on SMM involves 27 studies, with the change in fat‐free mass as a percentage of the change in total body mass ranging from 4% to 57%; with the fourth quartile showing 47% of the decrease in weight being due to a decrease in fat‐free mass [18]. This threefold to fourfold interstudy variability in measures of muscle mass loss implies that a subset of individuals treated with a GLP‐1RA, as with bariatric surgery, develop sarcopenia, suggesting this to indeed be a clinically meaningful consideration in a subset of treated persons.

Reduction in SMM with bariatric surgery is associated with measurable reductions in strength, as well as in walking speed and standing time [19, 20]. Our goal should be to develop clinically useful tools to follow individuals treated with GLP‐1RA to avoid the potential for increased mortality and for development of frailty with consequent functional deficits. A metabolite‐based measure would be clinically useful in the ascertainment of muscle mass and, in particular, in determining whether there are changes in muscle mass leading to potential development of sarcopenia.

The lysosomal proteinase inhibitor cystatin C is produced by all nucleated cells and present in all tissues and body fluids, while creatinine is particularly formed in skeletal muscle as a byproduct of the conversion of creatine to phosphocreatine. Both are cleared by the kidney, mainly by glomerular filtration, and have therefore been used as complementary approaches to estimating the glomerular filtration rate. The ratio of serum creatinine to cystatin C has been referred to as the sarcopenia index (SI). In a study of more than 450 000 UK Biobank participants, those with sarcopenia based on low maximum handgrip strength or reduction in muscle mass assessed using bioelectrical impedance had a 5%–10% lower SI, and those with both low handgrip strength and low muscle mass had a 13%–14% lower SI [21]. In > 15‐year followup of nearly 10 000 NHANES 1999–2004 participants, both total and CV mortality increased linearly with lower baseline SI levels [22]. Lower SI was associated with greater mortality in a meta‐analysis of 38 studies of > 20 000 hospitalized persons, as well as being associated with lower SMM, handgrip strength, and gait speed [23]. Among participants age > 75 years in the SPRINT intensive BP treatment trial, those in the lowest quintile of SI had nearly a 50% increase in CV events and in total mortality [24]. Among > 25 000 UK Biobank participants with diabetes not having diabetic microvascular complications at baseline, a measure similar to the SI was associated with greater likelihood of such complications, as well as with decreased muscle quantity and strength, and decreased functional status [25]. Finally, a study of 4635 persons aged ≥ 65 years showed that the more negative the difference between the estimated glomerular filtration rate (eGFR) estimated from cystatin C and that estimated from creatinine, the greater the likelihood of frailty as well as mortality [26], reflecting the inverse relationship both of creatinine and of cystatin C with the eGFR, and the positive relationship between serum creatinine and muscle mass. A caveat in the use of the SI in sarcopenic obesity is the evidence of association of obesity with higher cystatin C levels [27], so that a normalized creatinine to cystatin C ratio dividing by body weight has been proposed [28]. Measures of total and central obesity do not modify the association of cystatin C with CV or renal disease, or with mortality [29, 30], but specific study of the relationship between SI and sarcopenia in obesity is needed to confirm the validity of this measure. This may particularly be an issue with GLP‐1RA, as the reduction in fat mass with this treatment may lower cystatin C, leading to a higher SI without a corresponding improvement in muscle mass.

A recent report from a study of the effect of the combined GLP‐1 and glucose‐dependent insulinotropic polypeptide (GIP) receptor activator tirzepatide in patients with heart failure may shed light on some of these potential interactions. 731 persons age ≥ 40 with chronic heart failure, left ventricular ejection fraction ≥ 50% and BMI ≥ 30 randomized to tirzepatide versus placebo for a 52‐week period showed reduction in major heart failure events with improvement both in quality of life and in functional capacity measures, including 6‐min walk distance [31]. The authors remark on the association of the eGFR based on cystatin C with the eGFR based on creatinine, with “the 2 estimates var[ying] by as much as 50% in either direction, indicative of significant unexplained variance,” and also point out that the eGFR‐cystatin C was consistently and significantly ~9 mL/min/1.73 m^2^ lower than the eGFR‐creatinine [31]. In light of the above considerations, we can suggest that the lower eGFR‐creatinine in a population of persons with chronic heart failure reflects the lower muscle mass associated with sarcopenia in this group, and, furthermore, that the high person‐to‐person variability in the relationship between eGFR‐cystatin C and eGFR‐creatinine reflects interparticipant variability in degree of sarcopenia. Timed walking distance is a reasonable proxy in assessing sarcopenia [32, 33], similar to hand grip strength [34, 35]. Further analysis of this dataset to ascertain the relationship between 6‐min walking distance and the SI, both at baseline and after 52 weeks, would of interest in supporting the use of the SI in assessment of sarcopenia.

We can now propose a credible and testable hypothesis, and suggest an approach. First, the highly effective incretin receptor activators may be associated with the development of sarcopenia in one quarter or more of treated patients, with there being high interindividual variability in this effect, even in otherwise similar patients. Second, those individuals developing sarcopenia with this treatment may have greater likelihood of adverse outcome. Finally, approaches to prevent the development of sarcopenia can be utilized in patients treated with these agents, including incretin receptor activator dose reduction in appropriate patients, routine patient enrollment in structured exercise programs [36, 37], and ultimately the use of specific agents currently in development to prevent sarcopenia [38]. Incorporation of measures of sarcopenia is crucial for clinical trials of weight loss therapies, and the use of such approaches in clinical care should be strongly considered (Figure 1). The ratio of serum creatinine to cystatin C potentially offers a measure of sarcopenia which could be readily incorporated into clinical care.

**FIGURE 1:**
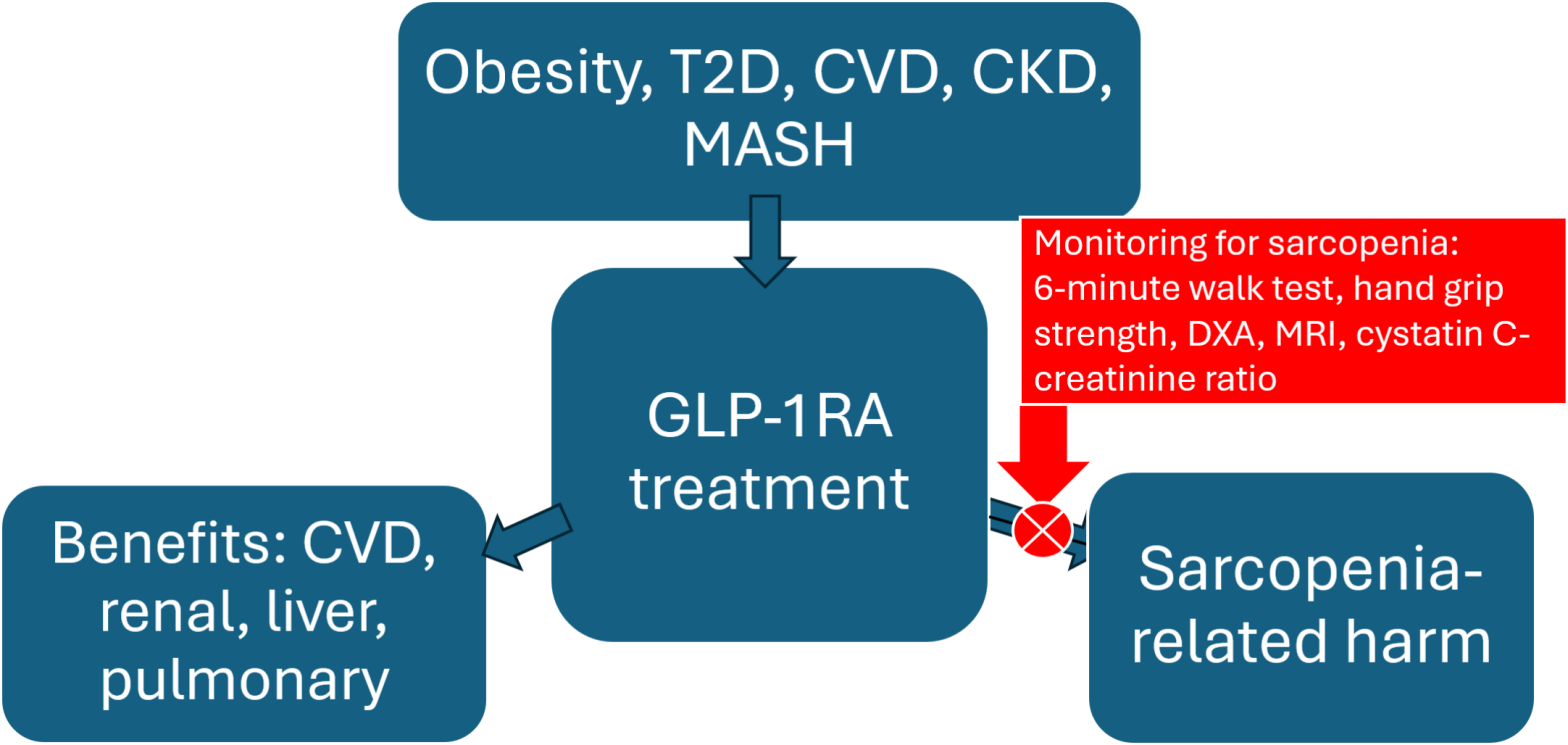
Incorporation of measures of sarcopenia into approaches to the use of incretin receptor agonists.

## Conflicts of Interest

The author declares no conflicts of interest.

## References

[1] World Obesity Federation , “World Obesity Atlas 2023,” https://data.worldobesity.org/publications/?cat=19.

[2] S. V. Badve , A. Bilal , M. M. Y. Lee , et al., “Effects of GLP‐1 Receptor Agonists on Kidney and Cardiovascular Disease Outcomes: A Meta‐Analysis of Randomised Controlled Trials,” Lancet Diabetes & Endocrinology 13, no. 1 (2025): 15–28. 39608381 10.1016/S2213-8587(24)00271-7

[3] Z. Bloomgarden , “Sarcopenia,” Journal of Diabetes 16, no. 10 (2024): e70025. 39470148 10.1111/1753-0407.70025 PMC11519986

[4] D. Gallagher , M. Visser , R. E. De Meersman , et al., “Appendicular Skeletal Muscle Mass: Effects of Age, Gender, and Ethnicity,” Journal of Applied Physiology 83, no. 1 (1997): 229–239. 9216968 10.1152/jappl.1997.83.1.229

[5] M. Xu , Y. Gong , and X. Yin , “Total and Regional Fat‐to‐Muscle Mass Ratio in Relation to All‐Cause and Cause‐Specific Mortality in Men and Women,” Journal of Clinical Endocrinology and Metabolism 110 (2024): e2054–e2063, 10.1210/clinem/dgae595. 39193721

[6] R. A. Joundi , B. Hu , S. Rangarajan , et al., “Activity Limitations, Use of Assistive Devices, and Mortality and Clinical Events in 25 High‐Income, Middle‐Income, and Low‐Income Countries: An Analysis of the PURE Study,” Lancet 404, no. 10452 (2024): 554–569. 39068950 10.1016/S0140-6736(24)01050-X

[7] A. Polo‐López , J. Calatayud , P. Palau , et al., “Joint Associations of Handgrip Strength and Physical Activity With Incident Cardiovascular Disease and Overall Mortality in the UK Biobank,” Clinical Nutrition 43, no. 12 (2024): 218–224. 10.1016/j.clnu.2024.10.022 39504675

[8] J. A. Batsis , T. A. Mackenzie , J. D. Jones , F. Lopez‐Jimenez , and S. J. Bartels , “Sarcopenia, Sarcopenic Obesity and Inflammation: Results From the 1999‐2004 National Health and Nutrition Examination Survey,” Clinical Nutrition 35, no. 6 (2016): 1472–1483. 27091774 10.1016/j.clnu.2016.03.028 PMC6432912

[9] I. Kaur , S. Das , S. Chandel , and S. Chandel , “Possible Sarcopenia, Sarcopenic Obesity Phenotypes and Their Association With Diabetes: Evidence From LASI Wave‐1 (2017‐18),” Diabetes and Metabolic Syndrome: Clinical Research and Reviews 19, no. 2 (2025): 103185, 10.1016/j.dsx.2025.103185. 39818062

[10] J. Linge , A. L. Birkenfeld , and I. J. Neeland , “Muscle Mass and Glucagon‐Like Peptide‐1 Receptor Agonists: Adaptive or Maladaptive Response to Weight Loss?,” Circulation 150, no. 16 (2024): 1288–1298. 39401279 10.1161/CIRCULATIONAHA.124.067676

[11] M. A. H. Nuijten , T. M. H. Eijsvogels , V. M. Monpellier , I. M. C. Janssen , E. J. Hazebroek , and M. T. E. Hopman , “The Magnitude and Progress of Lean Body Mass, Fat‐Free Mass, and Skeletal Muscle Mass Loss Following Bariatric Surgery: A Systematic Review and Meta‐Analysis,” Obesity Reviews 23, no. 1 (2022): e13370. 34664391 10.1111/obr.13370 PMC9285034

[12] KFF , “KFF Health Tracking Poll May 2024: The Public's Use and Views of GLP‐1 Drugs,” 2024, https://www.kff.org/health‐costs/poll‐finding/kff‐health‐tracking‐poll‐may‐2024‐the‐publics‐use‐and‐views‐of‐glp‐1‐drugs.

[13] J. P. H. Wilding , R. L. Batterham , S. Calanna , et al., “Once‐Weekly Semaglutide in Adults With Overweight or Obesity,” New England Journal of Medicine 384, no. 11 (2021): 989–1002. 33567185 10.1056/NEJMoa2032183

[14] C. Conte , K. D. Hall , and S. Klein , “Is Weight Loss‐Induced Muscle Mass Loss Clinically Relevant?,” JAMA 332 (2024): 9–10. 38829659 10.1001/jama.2024.6586

[15] B. A. Williams , “Perils of Evidence‐Based Medicine,” Perspectives in Biology and Medicine 53, no. 1 (2010): 106–120. 20173299 10.1353/pbm.0.0132

[16] S. R. Tunis , “Lack of Evidence for Clinical and Health Policy Decisions,” BMJ 347 (2013): f7155. 24343113 10.1136/bmj.f7155

[17] P. M. Rothwell , “External Validity of Randomised Controlled Trials: “to Whom Do the Results of This Trial Apply?”,” Lancet 365, no. 9453 (2005): 82–93. 15639683 10.1016/S0140-6736(04)17670-8

[18] R. L. Dubin , S. B. Heymsfield , E. Ravussin , and F. L. Greenway , “Glucagon‐Like Peptide‐1 Receptor Agonist‐Based Agents and Weight Loss Composition: Filling the Gaps,” Diabetes, Obesity and Metabolism 26, no. 12 (2024): 5503–5518. 10.1111/dom.15913 39344838

[19] D. L. Alba , L. Wu , P. M. Cawthon , et al., “Changes in Lean Mass, Absolute and Relative Muscle Strength, and Physical Performance After Gastric Bypass Surgery,” Journal of Clinical Endocrinology and Metabolism 104, no. 3 (2019): 711–720. 30657952 10.1210/jc.2018-00952 PMC6339456

[20] P. Ibacache‐Saavedra , E. Martínez‐Rosales , D. Jerez‐Mayorga , C. Miranda‐Fuentes , E. G. Artero , and M. Cano‐Cappellacci , “Effects of Bariatric Surgery on Muscle Strength and Quality: A Systematic Review and Meta‐Analysis,” Obesity Reviews 25, no. 9 (2024): e13790. 38859617 10.1111/obr.13790

[21] T. J. Wilkinson , L. A. Baker , E. L. Watson , A. C. Smith , and T. Yates , “Diagnostic Accuracy of a ‘sarcopenia Index’ Based on Serum Biomarkers Creatinine and Cystatin C in 458,702 UK Biobank Participants,” Clinical Nutrition ESPEN 63 (2024): 207–213. 38968079 10.1016/j.clnesp.2024.06.041

[22] T. Wang , Y. Zhu , X. Liu , et al., “Cystatin C and Sarcopenia Index Are Associated With Cardiovascular and All‐Cause Death Among Adults in the United States,” BMC Public Health 24, no. 1 (2024): 1972. 39044229 10.1186/s12889-024-19137-x PMC11267836

[23] W. H. Zheng , Y. B. Zhu , Y. Yao , and H. B. Huang , “Serum Creatinine/Cystatin C Ratio as a Muscle Mass Evaluating Tool and Prognostic Indicator for Hospitalized Patients: A Meta‐Analysis,” Frontiers in Medicine 9 (2023): 1058464. 36698829 10.3389/fmed.2022.1058464 PMC9868859

[24] S. Mirzai , I. Persits , R. Kazibwe , et al., “Relationship Between Sarcopenia and Intensive Blood Pressure Control Efficacy and Safety: A Secondary Analysis of SPRINT,” Hypertension 81, no. 8 (2024): e77–e87. 38881460 10.1161/HYPERTENSIONAHA.124.23011 PMC11254568

[25] D. He , B. Gao , J. Wang , C. Yang , M. H. Zhao , and L. Zhang , “The Difference Between Cystatin C‐ and Creatinine‐Based Estimated Glomerular Filtration Rate and Risk of Diabetic Microvascular Complications Among Adults With Diabetes: A Population‐Based Cohort Study,” Diabetes Care 47, no. 5 (2024): 873–880. 38470988 10.2337/dc23-2364 PMC11043223

[26] O. A. Potok , R. Katz , N. Bansal , et al., “The Difference Between Cystatin C‐ and Creatinine‐Based Estimated GFR and Incident Frailty: An Analysis of the Cardiovascular Health Study (CHS),” American Journal of Kidney Diseases 76, no. 6 (2020): 896–898. 32682698 10.1053/j.ajkd.2020.05.018 PMC7967899

[27] P. Muntner , J. Winston , J. Uribarri , D. Mann , and C. S. Fox , “Overweight, Obesity, and Elevated Serum Cystatin C Levels in Adults in the United States,” American Journal of Medicine 121, no. 4 (2008): 341–348. 18374694 10.1016/j.amjmed.2008.01.003 PMC3049932

[28] H. Sun , Z. Wu , G. Wang , and J. Liu , “Normalized Creatinine‐To‐Cystatin C Ratio and Risk of Cardiometabolic Multimorbidity in Middle‐Aged and Older Adults: Insights From the China Health and Retirement Longitudinal Study,” Diabetes and Metabolism Journal 49 (2025): 448–461, 10.4093/dmj.2024.0100. 39829108 PMC12086583

[29] S. S. Panaich , V. Veeranna , C. Bavishi , S. K. Zalawadiya , A. Kottam , and L. Afonso , “Association of Cystatin C With Measures of Obesity and Its Impact on Cardiovascular Events Among Healthy US Adults,” Metabolic Syndrome and Related Disorders 12, no. 9 (2014): 472–476. 25118891 10.1089/met.2014.0018

[30] D. C. Chen , R. Scherzer , J. H. Ix , et al., “Modification of Association of Cystatin C With Kidney and Cardiovascular Outcomes by Obesity,” American Journal of Kidney Diseases 83, no. 4 (2024): 489–496.e1. 37866793 10.1053/j.ajkd.2023.08.021 PMC10960714

[31] M. Packer , M. R. Zile , C. M. Kramer , et al., “Interplay of Chronic Kidney Disease and the Effects of Tirzepatide in Patients With Heart Failure, Preserved Ejection Fraction, and Obesity: The SUMMIT Trial,” Journal of the American College of Cardiology 85, no. 18 (2025): 1721–1735. 40162940 10.1016/j.jacc.2025.03.009

[32] C. Steinmetz , L. Krause , S. Sulejmanovic , et al., “The Prevalence and Impact of Sarcopenia in Older Cardiac Patients Undergoing Inpatient Cardiac Rehabilitation ‐ Results From a Prospective, Observational Cohort Pre‐Study,” BMC Geriatrics 24, no. 1 (2024): 94. 38267843 10.1186/s12877-024-04694-y PMC10809534

[33] J. Woo , J. Leung , A. Sham , and T. Kwok , “Defining Sarcopenia in Terms of Risk of Physical Limitations: A 5‐Year Follow‐Up Study of 3,153 Chinese Men and Women,” Journal of the American Geriatrics Society 57, no. 12 (2009): 2224–2231. 19925615 10.1111/j.1532-5415.2009.02566.x

[34] Y. C. Ha , S. C. Hwang , S. Y. Song , C. Lee , K. S. Park , and J. I. Yoo , “Hand Grip Strength Measurement in Different Epidemiologic Studies Using Various Methods for Diagnosis of Sarcopenia: A Systematic Review,” European Geriatric Medicine 9, no. 3 (2018): 277–288. 34654240 10.1007/s41999-018-0050-6

[35] J. I. Yoo , H. Choi , and Y. C. Ha , “Mean Hand Grip Strength and Cut‐Off Value for Sarcopenia in Korean Adults Using KNHANES VI,” Journal of Korean Medical Science 32, no. 5 (2017): 868–872. 28378563 10.3346/jkms.2017.32.5.868 PMC5383622

[36] D. T. Villareal , S. Chode , N. Parimi , et al., “Weight Loss, Exercise, or Both and Physical Function in Obese Older Adults,” New England Journal of Medicine 364, no. 13 (2011): 1218–1229. 21449785 10.1056/NEJMoa1008234 PMC3114602

[37] J. R. Lundgren , C. Janus , S. B. K. Jensen , et al., “Healthy Weight Loss Maintenance With Exercise, Liraglutide, or Both Combined,” New England Journal of Medicine 384, no. 18 (2021): 1719–1730. 33951361 10.1056/NEJMoa2028198

[38] A. A. Sayer , R. Cooper , H. Arai , et al., “Sarcopenia,” Nature Reviews Disease Primers 10, no. 1 (2024): 68. 10.1038/s41572-024-00550-w 39300120

